# Study on Structure, Thermal Behavior, and Viscoelastic Properties of Nanodiamond-Reinforced Poly (vinyl alcohol) Nanocomposites

**DOI:** 10.3390/polym13091426

**Published:** 2021-04-28

**Authors:** Tomáš Remiš, Petr Bělský, Tomáš Kovářík, Jaroslav Kadlec, Mina Ghafouri Azar, Rostislav Medlín, Veronika Vavruňková, Kalim Deshmukh, Kishor Kumar Sadasivuni

**Affiliations:** 1 New Technologies—Research Centre, University of West Bohemia, Univerzitní 8, 306 14 Plzeň, Czech Republic; pbelsky@ntc.zcu.cz (P.B.); toko@ntc.zcu.cz (T.K.); jakadlec@ntc.zcu.cz (J.K.); minag@kmm.zcu.cz (M.G.A.); medlin@ntc.zcu.cz (R.M.); vavrunko@ntc.zcu.cz (V.V.); deshmukh@ntc.zcu.cz (K.D.); 2 Center for Advanced Materials, Qatar University, Doha P.O. Box 2713, Qatar; kishorkumars@qu.edu.qa

**Keywords:** poly (vinyl alcohol), nanodiamond, nanocomposite, mechanical properties, morphology

## Abstract

In this work, advanced polymer nanocomposites comprising of polyvinyl alcohol (PVA) and nanodiamonds (NDs) were developed using a single-step solution-casting method. The properties of the prepared PVA/NDs nanocomposites were investigated using Raman spectroscopy, small- and wide-angle X-ray scattering (SAXS/WAXS), scanning electron microscopy (SEM), transmission electron microscopy (TEM), thermogravimetric analysis (TGA), differential scanning calorimetry (DSC), and dynamic mechanical analysis (DMA). It was revealed that the tensile strength improved dramatically with increasing ND content in the PVA matrix, suggesting a strong interaction between the NDs and the PVA. SEM, TEM, and SAXS showed that NDs were present in the form of agglomerates with an average size of ~60 nm with primary particles of diameter ~5 nm. These results showed that NDs could act as a good nanofiller for PVA in terms of improving its stability and mechanical properties.

## 1. Introduction

Nowadays, polymeric materials are being used in a broad range of fields such as aviation, sports products, automobiles, and electronic devices. Such applications utilize the advantageous features of polymeric materials, including low specific weight, electrical insulation, tribological properties, and flexibility. Poly (vinyl alcohol; PVA) is a water-soluble commodity polymer, which exhibits good tensile strength and flexibility, excellent film-forming characteristics, good adhesive and emulsifying properties, biocompatibility, high water uptake, and non-carcinogenicity. Furthermore, PVA found applications in various industries, including textile manufacturing, papermaking and processing, packaging, gastronomy, and particularly, biomedical and pharmaceutical industries [1,2,3,4]. The properties such as high water uptake, elasticity, and biocompatibility make PVA a potential candidate for tissue engineering [5,6,7]. Moreover, PVA was investigated as contact lenses, synthetic heart covers, artificial cartilage, catheters, skin, and pancreas membranes [8,9,10,11,12,13,14,15]. Because of its biocompatibility, drug integration, solubility, film-forming capability, and good stretchability and swelling properties, PVA was studied as drug delivery systems in the oral, transdermal, intramuscular, and rectal ways. The degree of crystallinity of PVA was very important in the diffusion of the drugs from the films [16,17,18]. PVA films have good swelling characteristics, which was beneficial for several research fields. Some studies tried to enhance the low thermal stability of PVA-based polymers with various material modifications, e.g., cross-linking [19,20], electrospinning [21,22,23], blending [24], sol-gel processing [25,26], and grafting [27,28]. Cross-linking is considered as an appropriate method to adjust the physical and chemical properties of PVA films, which can be performed with chemicals able to react with –OH groups [29]. For instance, Maitra et al. [30] and Morimune et al. [31] cross-linked the PVA matrix by incorporation of nanodiamond (ND) particles with a concentration of 0–5 wt% (the size of the ND particles was up to 10 nm and size of ND agglomerates was 100–200 nm) which led to an enhancement of its mechanical properties of almost 2.5 times.

Considerable scientific attention has been directed to NDs owing to their special shape and chemical, biological, mechanical, and optical properties. The main advantages of ND particles are seen in their excellent strength, stiffness, good thermal conductivity and electrical resistivity, optical properties and fluorescence, low coefficient of friction, chemical stability, and resistance to harsh environments, and biocompatibility [32,33,34,35,36,37]. The small and uniform size of elementary ND particles, with an almost spherical structure, is considered a great benefit because their distribution (intermixing) in the material is better compared to, for example, carbon nanotubes and graphene. In addition, an interesting advantage seems to be the possibility of setting their particle size distribution; they can be separated into basic particles (nm sized) or be present as aggregates of these particles (tens to hundreds of nm), or as a mixture of basic particles and aggregates of different size and in this way, it is possible to control their functionality in the composite system. What is also beneficial is their extensive and accessible surface, optimized interactions with the matrix, i.e., high interphase volume, and the rich and adaptable surface chemistry, providing high adaptability for the ND–matrix interface [38,39]. Moreover, NDs are not toxic, which makes them appropriate for biomedical uses. As a result, these nanomaterials have a great application potential in drug delivery [40,41,42,43,44,45,46,47,48], bioimaging [49,50,51,52], tribology and lubrication [53,54,55], protein mimics [56,57,58], and tissue engineering [59,60].

In the present study, a solution-casting method was applied to produce PVA-ND nanocomposites. Here we presented the first thorough study combining the analyses of thermal behavior, viscoelastic properties, and structure of PVA-ND nanocomposites with varying ND contents. The PVA-ND nanocomposites were investigated using Raman spectroscopy, SAXS/WAXS, SEM, TEM, TGA, DSC, and DMA for this purpose.

## 2. Materials and Methods

### 2.1. Materials

PVA (hydrolyzed 99+%, Mw = 86,000–98,000 g/mol) was purchased from Sigma-Aldrich, USA. Detonation ND powder of grade G01 was obtained from Plasma Chem GmbH, Germany. In-house prepared deionized (DI) water, with a resistivity of 14.3 MΩ·cm 25 °C, was utilized for the preparation of nanocomposites.

### 2.2. Nanocomposite Preparation

**Figure 1:**
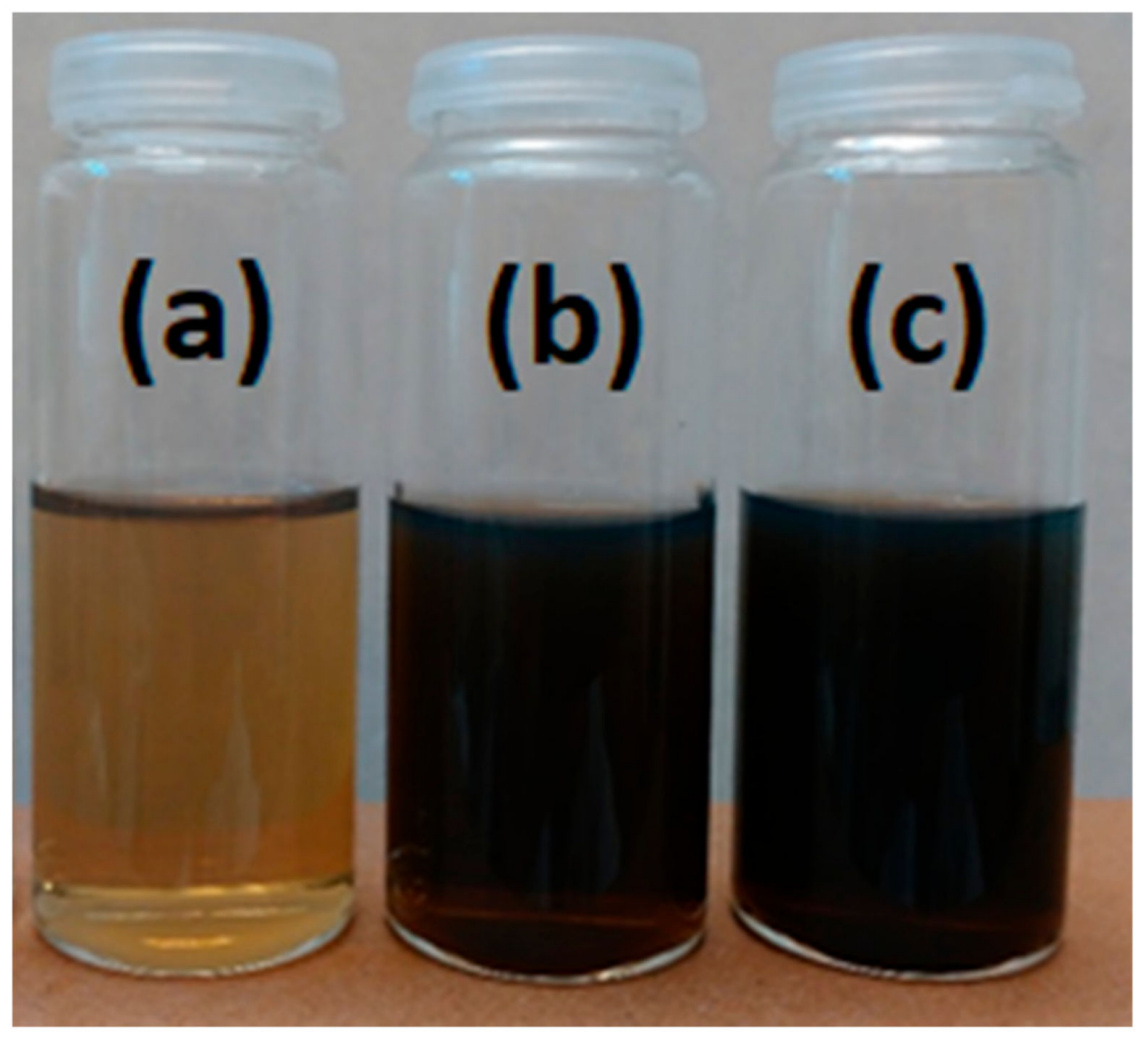
Visual comparison of the ND dispersions at the three weight concentrations, (**a**) 0.25, (**b**) 1, and (**c**) 1.9 wt% in DI water.

The PVA-ND nanocomposites were prepared by a solution casting method. NDs were prepared according to the synthesis protocol previously published by Kovářík et al. [61]. The light scattering studies of ND dispersions revealed that the mean size of ND aggregates in the dispersions was between ~61 and 73 nm and that the mean size did not show any systematic dependence on the ND concentration [61]. The NDs were heat treated (annealing at 450 °C for 3 h in an air atmosphere) to supply them with a negative surface charge to activate the surface chemistry. The surface charge of the nanoparticles obtained from the zeta potential measurement was −52 mV. Then, the annealed ND powder was dispersed in DI water at various concentrations, as shown in Table 1. An ultrasonic homogenizer, Sonopuls HD 3200 (BANDELIN Electronic GmbH, Germany), was used to achieve the appropriate dispersion of NDs. Sonication for 3 h with an operating frequency of 20 kHz and an energy conversion of 400 kJ and, with continuous stirring, was used to obtain a brown-to-black-colored nanodispersion (Figure 1). After that, the dispersions were centrifuged 3× at 4000 rpm (2325 g) for 1 h. Next, a 5 wt% solution of PVA in the NDs dispersions was completed by continuous mixing for ~2 h at 90 °C until all PVA was dissolved. The completed dispersions were then formed by the casting machine (AB3120, TQC Sheen Co., Germany) with a glass surface to fabricate nanocomposite films, followed by drying at 40 °C overnight. The prepared films had a uniform thickness of 49.3 μm (±5.3 μm) measured using a micrometer. The composition of the dispersions and the prepared nanocomposite films are given in Table 1.

### 2.3. Characterization

The Raman measurements of the nanocomposite films were carried out on a DXR Raman microscope (Thermo Fisher Scientific Inc., Bartlesville, OK, USA). Further, a 532 nm excitation laser with a 50× objective was used. The laser power was 10 mW, and 100 scans with a 1s exposure time were taken for every spectrum.

The SAXS/WAXS measurements were carried out on a SAXSess mc^2^ instrument (Anton Paar GmbH, Graz, Austria) which is a Kratky-type instrument consisting of a microfocus X-ray source with a Cu anode, single reflection focusing X-ray optics, and a collimation block. Image plates were used for X-ray detection; the image plates were read out using the CyclonePlus^®^ Reader (Perkin Elmer, Inc., Boston, USA). The range of the magnitude of the scattering vector, *q*, was 0.2 nm^−1^–28 nm^−1^, i.e., both SAXS and WAXS were measured at the same time. The *q* is given as $q=\frac{4\pi }{\lambda }\sin\theta$; *λ* is the X-ray wavelength (0.154 nm for Cu Kα) and *θ* is the half scattering angle. The exposure time was 30 min, and several pieces of the films were stacked onto each other in each experiment to increase the sample thickness and, thus, also the scattering intensity. The 1D radial intensity profiles were calculated from the obtained 2D patterns by azimuthal averaging using SAXSquant^TM^ software supplied with the instrument. It was possible to perform this procedure thanks to the azimuthal symmetry of the scattering patterns (which indicated that the investigated material was isotropic). Both the correction for sample transmittance and the correction for the background scattering (with an empty sample holder) were carried out. The profiles were also managed for film thickness. Finally, the incoherent scattering background (taken as constant and equal to the scattering intensity at about 7 nm^−1^) was subtracted. For the presentation of WAXS results, the scattering vector magnitude scale was converted to a *2θ* scale. The Irena program for SAXS evaluations [62] was used for the quantification of the nanoscale morphology characteristics. For the analysis of the ND morphology, the unified exponential/power-law approach [63] was applied in the Irena software.

The structure of the PVA-ND nanocomposite films was studied by Field Emission Scanning Electron Microscope (FE-SEM, JSM-7600F, JEOL Ltd., Tokyo, Japan). The gentle beam mode was used to minimize the sample charging and the adverse electron beam effects on the samples. 

In this, a negative voltage was applied to the specimen holder, which decelerated the incident electrons just before they irradiated the specimen; thus, the resolution was improved at an extremely low accelerating voltage. The morphologies of the ND dispersions were analyzed with TEM (JEM-2200FS, JEOL Ltd., Tokyo, Japan).

A Q500 TGA analyzer (TA Instruments Corp., New Castle, DE, USA) was used to determine the thermal stability of the various PVA-ND nanocomposite films. The temperature rose at a rate of 10 °C/min. N_2_ atmosphere with a flow of 60 mL/min was used. The weight of the samples was 3.4 mg, 3.9 mg, 5.5 mg, and 5.9 mg for PVA, PVA-ND/1, PVA-ND/2, and PVA-ND/3, respectively. 

Differential scanning calorimetry analyses of the nanocomposites were done on a Q200 DSC analyzer (TA Instruments Corp., New Castle, USA). The samples were placed in the aluminum pans, and linear heating scans were carried out from 30 to 400 °C at a heating rate of 10 °C/min in an N_2_ atmosphere. The weight of the samples was 1.4 mg, 1.6 mg, 1.3 mg, and 1.3 mg for PVA, PVA-ND/1, PVA-ND/2, and PVA-ND/3, respectively.

The tensile properties and the temperature dependence of tan delta were determined by a Q800 DMA instrument (TA Instruments Corp., New Castle, USA). The initial length of the specimen for tensile measurements was 20 mm (6 mm wide), and the extension rate was 1 mm/min. For measurement, geometry was used for axial testing for film/fiber tension. The measurements were carried out at room temperature. Samples, 20 mm long and 6 mm wide, were used to measure the thermomechanical properties (tan delta). The experiments were performed in an N_2_ atmosphere at a frequency of 1 Hz starting at room temperature up to 80 °C, with a heating rate of 5 °C/min. 

## 3. Results and Discussions

### 3.1. Raman Spectroscopy

**Figure 2:**
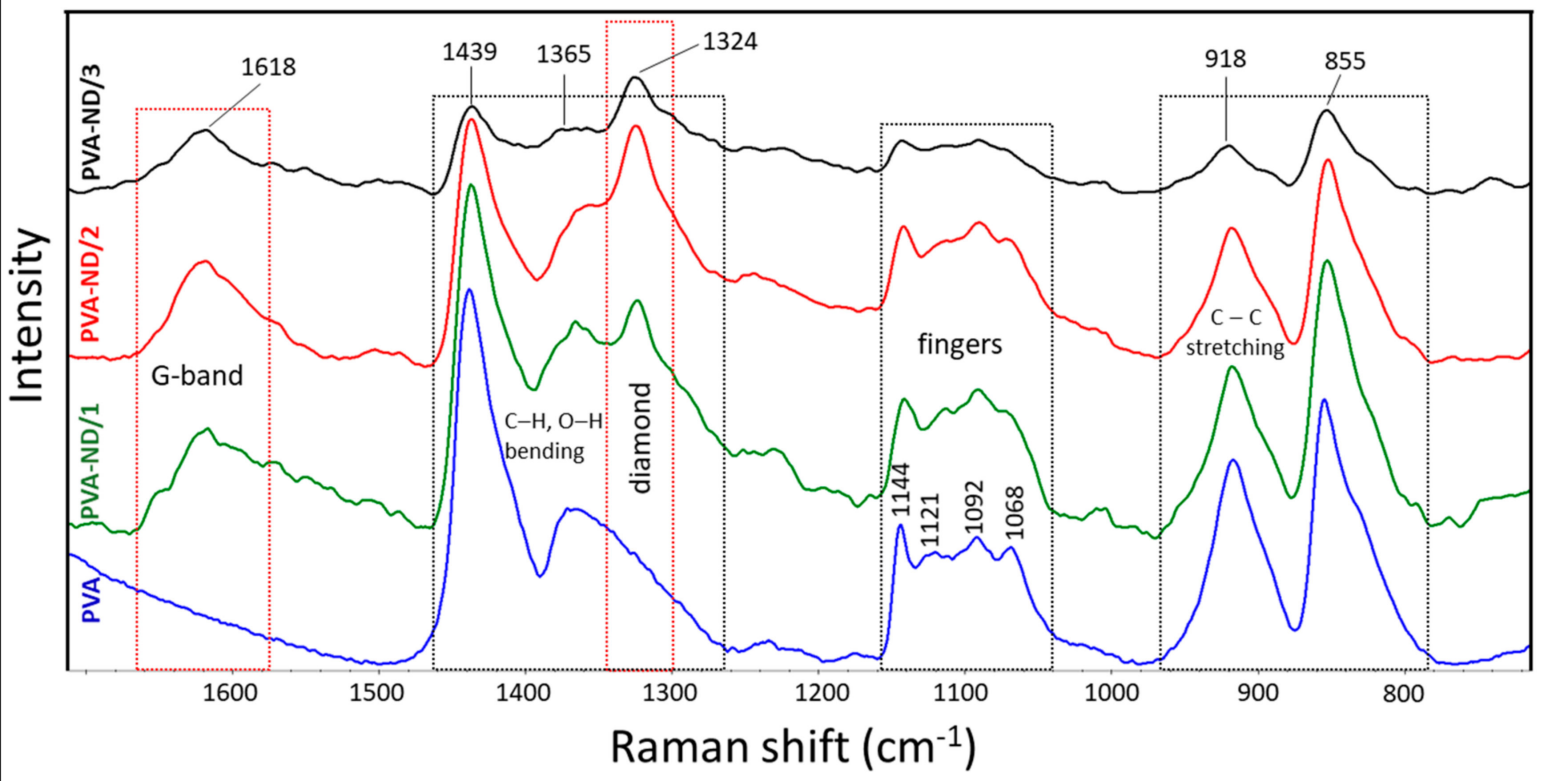
Raman spectra of the PVA and the PVA-NDs nanocomposite films.

The Raman spectra of the PVA and the PVA-ND nanocomposites were investigated. The strong characteristic bands of PVA were observed at 855, 918, 1365, and 1439 cm^−1^ (Figure 2). Further typical bands of PVA were observed at 1068, 1092, 1121, and 1144 cm^−1^. The bands at 855 (crystalline) and 918 cm^−1^ (amorphous) resulted from the C–C stretching of the PVA carbon backbone in the crystalline phase and the amorphous phase, respectively. The bands at 1365 and 1439 cm^−1^ were assigned to O–H and C–H bending vibrations, respectively, and 1068 and 1092 cm^−1^ corresponded to C–O stretching and O–H bending, respectively. The next bands at 1121 and 1144 cm^−1^ indicated the C–O and C–C stretching in the crystalline phase of the PVA matrix [64,65,66,67].

**Scheme 1:**
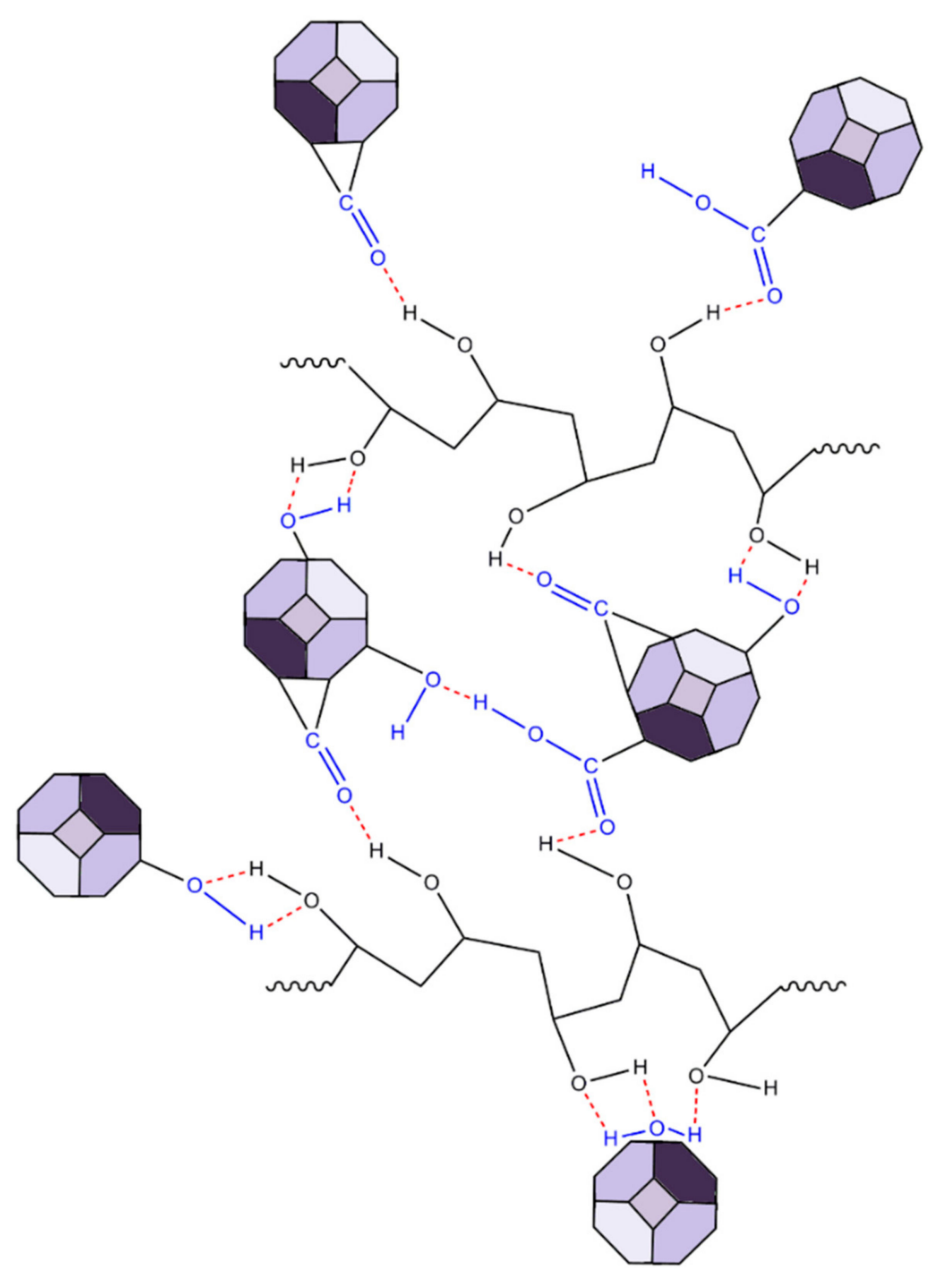
Model scheme of the PVA and ND nanocomposite structure.

The ND-containing PVA nanocomposites exhibited two strong characteristic bands at 1324 and 1618 cm^−1^, which were not present in the Raman spectra of PVA. More specifically, the band at 1324 cm^−1^ was characteristic of sp3 carbon bonding (the diamond band), and that at 1618 cm^−1^ (G-band) was typical for graphite or sp2 bonding [68,69]. The intensity of the diamond band (1324 cm^−1^) and the G-band (1618 cm^−1^) increased with the ND content. For the PVA-ND/3 film, the intensity was reduced due to the high concentration of NDs in the matrix (28%), causing the shading of the bonds owing to the nanodiamond particles. Furthermore, the crystalline bands of PVA at 1144 cm^−1^, 1121 cm^−1^, and 855 cm^−1^ decreased with increasing ND content to a greater extent than the decrease in the relative amount of PVA, particularly for the PVA-ND/3 sample, which indicated a decrease in the PVA crystallinity—fewer polymer chains were participating in this stretching. This corresponded well with the WAXS and DSC results, as discussed in Section 3.3 and 3.6, respectively. The decrease in the 918 cm^−1^ and 855 cm^−1^ bands was due to the replacement of the C–C bonds of the PVA backbone by the hydrogen bonds between the NDs and the PVA. The oxygen-containing functional groups (CO, COOH, OH) on the surface of the NDs participated (see Scheme 1) to create strong interaction, including hydrogen bonding between the NDs and the PVA while restricting the PVA’s crystallization [31]. The proposed interactions between PVA and ND particles are illustrated in Scheme 1.

### 3.2. SAXS/WAXS

**Figure 3:**
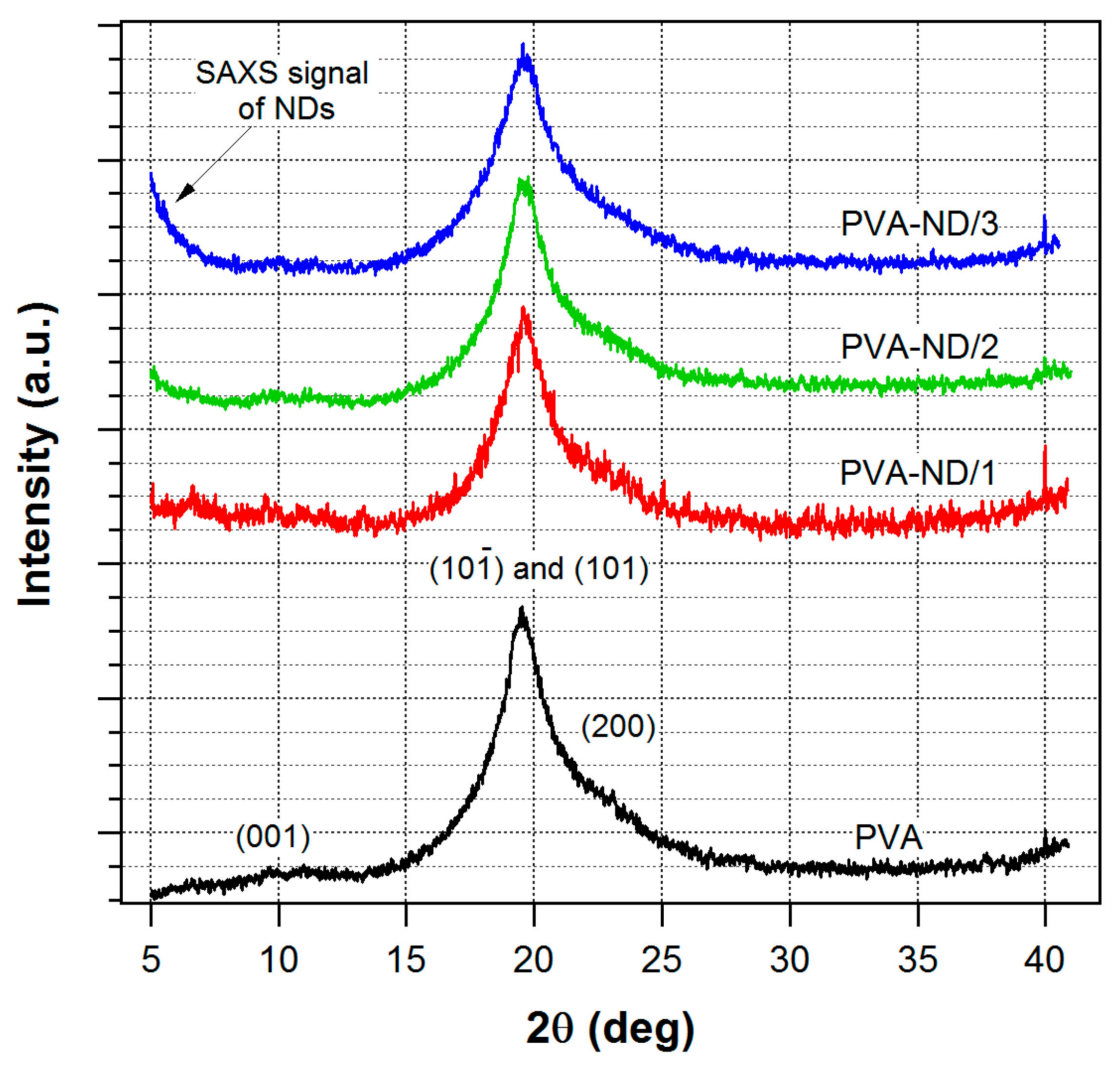
WAXS profiles of the pure PVA film and the nanocomposites.

The WAXS profile of the pure PVA film and the nanocomposite samples (Figure 3) showed one major and two minor reflections that were assigned as reflections of the monoclinic structure of PVA [70,71]. The peaks were relatively wide, indicating that the crystallite size was very small. The profiles of the pure PVA sample and the PVA nanocomposite samples did not differ significantly, which indicated that the morphology of the PVA was not substantially changed by the incorporation of NDs. After a detailed look, only a slight change could be noticed in Figure 3. With the increasing amount of ND in the sample, the main PVA peak, its FWHM, got somewhat broader (mainly for PVA-ND3), indicating that the high ND concentration disturbed the formation of the PVA crystallites to some extent. 

**Figure 4:**
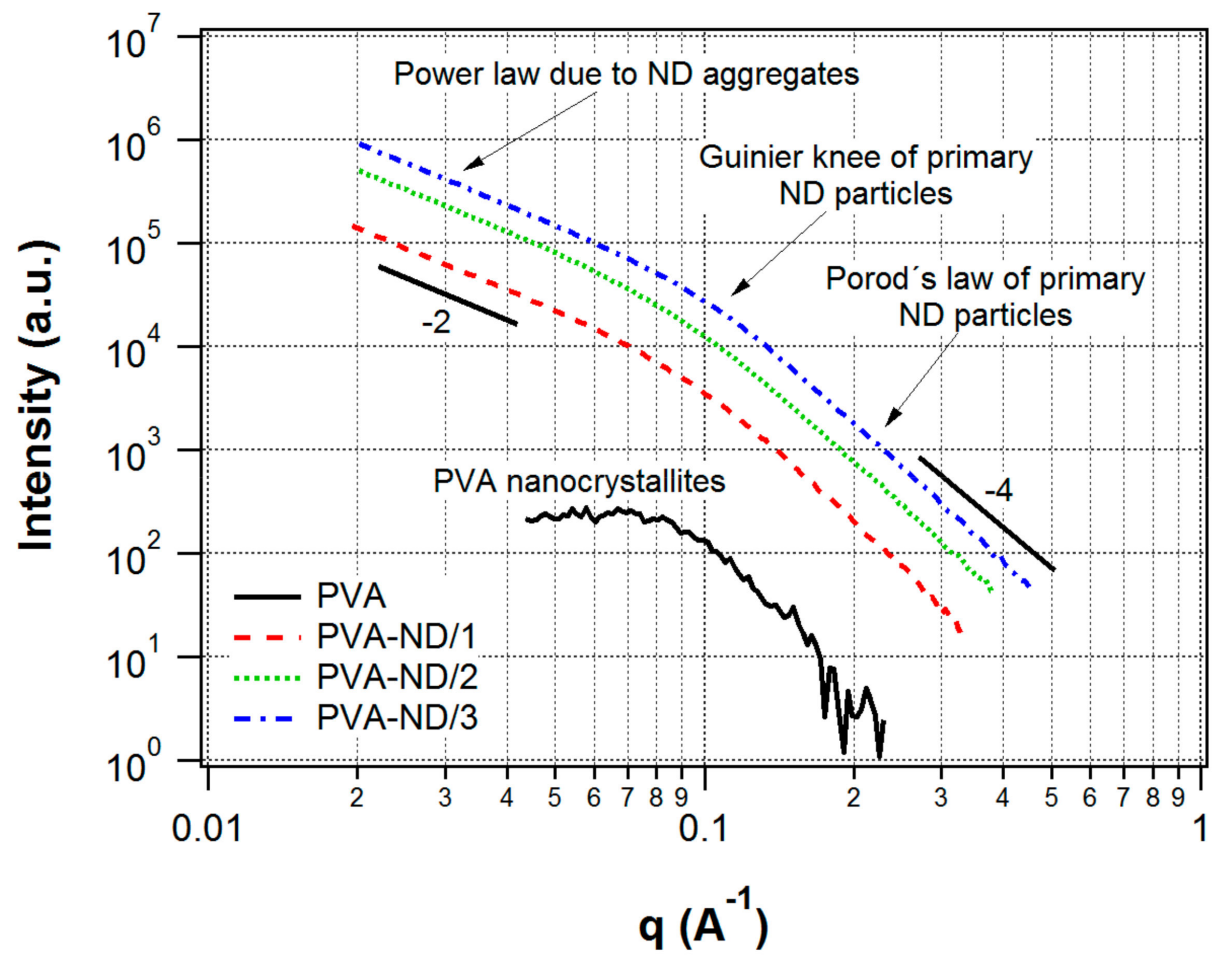
SAXS profiles of the pure PVA film and the nanocomposites.

The shoulder, which could be observed in the SAXS profile of the pure PVA film (Figure 4), indicated that nanostructures were present in the PVA film. Considering the discussed WAXS results for PVA, i.e., the detected crystallinity, the SAXS signal was ascribed to nanocrystallites of PVA. It was a confirmation of the semicrystalline nature of PVA, which was composed of nanocrystallites embedded in an amorphous matrix [8]. The model fitting in Irena software showed an average size of the nanoscale crystallites of 4.5 nm.

In contrast to WAXS, the SAXS profiles of the nanocomposites (Figure 4) were totally different compared to pure PVA. The SAXS intensities were orders of magnitude higher than in the case of the pure PVA film, and they showed a profile typical for ND agglomerates [72]. The reason for the difference in SAXS profiles of pure PVA vs. nanocomposites was that the presence of NDs in PVA led to a much stronger scattering in the SAXS than the scattering due to the PVA nanocrystallites in the PVA amorphous matrix and, thus, the scattering of the PVA nanocrystallites was negligible in the strong scattering originating from the ND particles. Moreover, the SAXS profiles of the nanocomposites did not differ substantially from the scattering profiles of the initial NDs dispersions used for the preparation of the films prepared in this work. These dispersions were studied in our previous work [61]. Thus, the structure of the ND aggregates did not substantially change after the addition into the PVA.

The SAXS profiles of the nanocomposites were interpreted as originating from ND fractal agglomerates. The fractal agglomerates were irregular particle accumulations displaying self-similarity on a specific interval of length scales. The fundamental particles that form the agglomerate were denoted as primary particles [73]. A mass fractal was characterized by a decreasing density from the agglomerate center to the edges, and its mass scales with the *d_f_*-th power of its radius, $m\propto r^{d_{f}}$ [74], where *d_f_* is the fractal dimension. In general, the fractal dimension is a non-integer (1 < *d_f_* < 3). Thus, a fractal agglomerate could be thought of as an object of non-integer dimensionality, although, of course, it is a 3D object in general. The higher the fractal dimension, the more branched and denser was the structure of the fractal agglomerate. Fractal agglomerates were formed in diverse materials, e.g., silica, gold, some polymeric materials, etc. For the investigation of their morphology, small-angle scattering techniques were one of the best-suited tools [74,75]. It is known that ND dispersions also show the mass-fractal structure [72,76]. 

The observed profiles of our nanocomposites showed one knee (Guinier region) pertaining to the ND primary particles. On the right side of this knee, Porod’s region ($I\propto q^{-4}$) ascribed to the ND primary particles could be observed. On the left side of the Guinier knee, there was a power-law dependence ($I\propto q^{-d_{f}}$) with an exponent of approximately −2 (thus, *d_f_* ~ 2), which was due to the agglomerates. A second Guinier knee pertaining to the agglomerates was not seen—it would be found at lower *q*-values, which were out of the range of the instrument used.

The model fitting of the nanocomposites SAXS profiles with the Unified Fit tool in the Irena program, based on the unified exponential/power-law approach [63], provided a radius of gyration (*R_g_*) of 1.9–2.0 nm, corresponding to ND primary particles of average particle size 4.8–5.1 nm (assuming the ND particles were homogeneous spheres). The fractal dimension of the agglomerates, *d_f_*, was approximately 1.9–2.0, based on the model fitting. In our previous work on ND dispersions, the second Guinier knee, corresponding to the agglomerates, was observed in Ultra-Small-Angle X-ray Scattering (USAXS) profiles (obtained at the APS synchrotron) [61]. The same ND dispersions were used for the preparation of the PVA-ND nanocomposites in this work. The agglomerate sizes in the initial dispersions were about 60 nm based on the USAXS results. However, the PVA-ND nanocomposites presented here were not investigated with USAXS due to our limited access to the synchrotron facility.

### 3.3. TEM and FE-SEM

**Figure 5:**
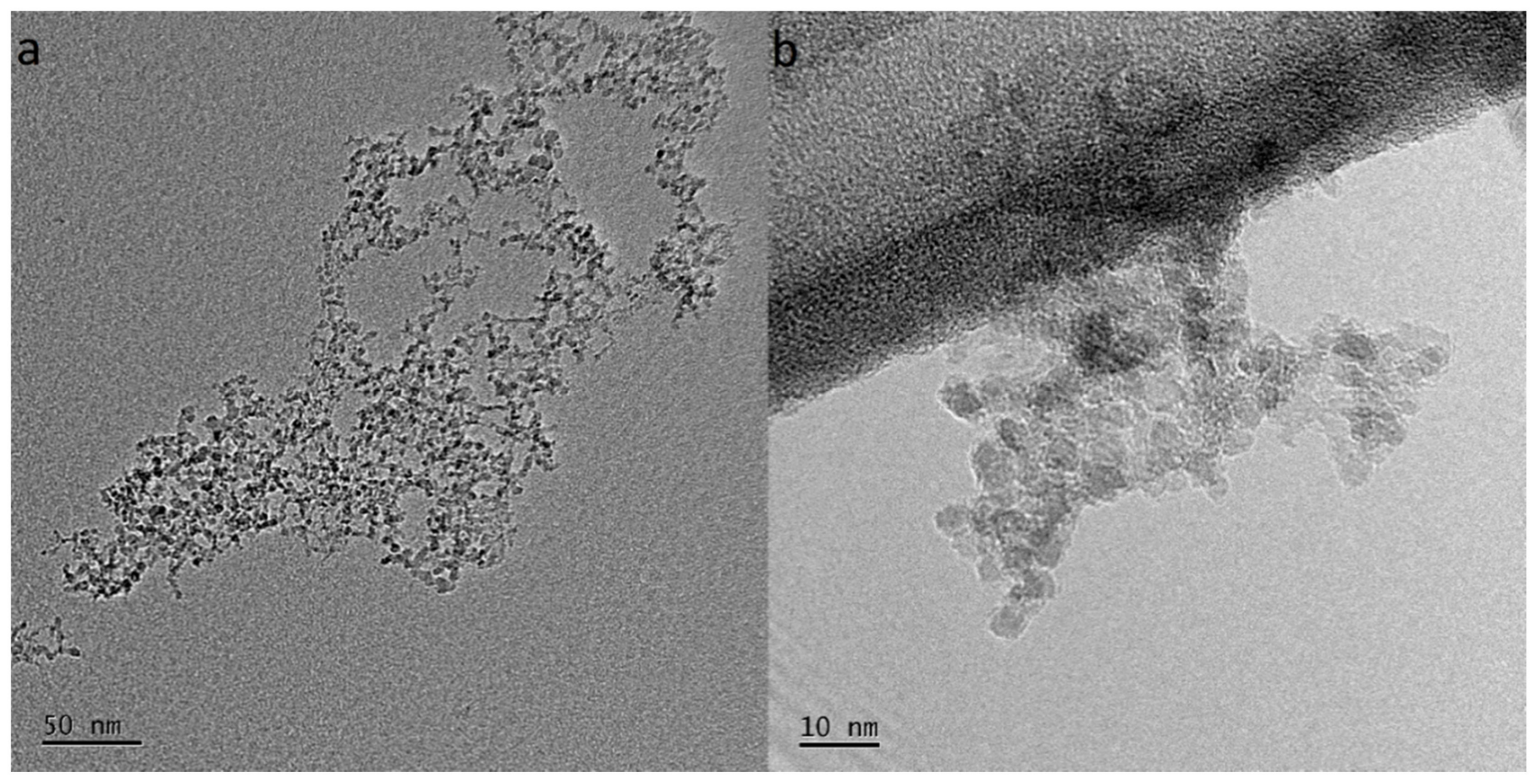
TEM images of the agglomerates of NDs dispersed in water. The size of the agglomerates in (**a**) was 50–150 nm, and the size of the ND primary particles in (**b**) was ~5 nm.

To observe the morphology, size, and spatial distribution of the NDs dispersed in the DI water, TEM microscopy was performed. Figure 5 shows bright-field images consisting of numerous nanoparticles grouped into agglomerates. The size of the agglomerates was in the range of 50–150 nm, while the primary ND particle size being ~5 nm.

**Figure 6:**
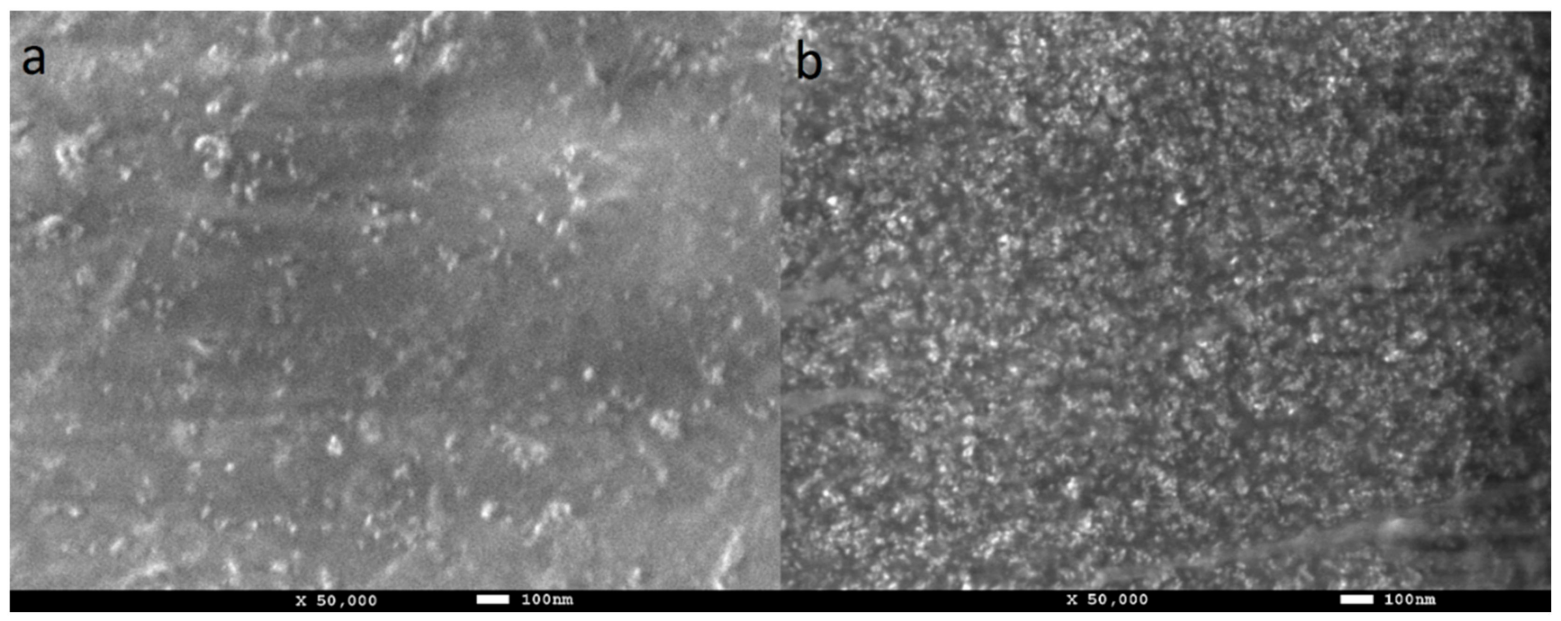
SEM photographs of the cast film surfaces of (**a**) PVA-ND/1 and (**b**) PVA-ND/3 nanocomposites.

The morphology of the PVA-ND nanocomposites was investigated using FE-SEM (Figure 6). It was evident that the ND agglomerates were well dispersed in the PVA matrix. The size of the agglomerates observed with FE-SEM was in good agreement with the TEM measurement (Figure 5) and the USAXS measurements [61], both of which showed agglomerate sizes around 60 nm. The increase in the ND concentration in PVA-ND/3 (Figure 6b) was obvious in comparison with PVA-ND/1 (Figure 6a). The ND concentrations in the PVA-ND/1 and PVA-ND/3 dried films were for ~5 wt% and ~28 wt%, respectively, as given in Table 1. 

Therefore, the results of Raman spectroscopy, SEM and TEM, suggested that the increased amount of oxygen-containing functional groups, due to the thermo-oxidative treatment of NDs, effectively facilitated their dispersion in the PVA matrix and incorporation into the polymer structure. 

### 3.4. TGA

**Figure 7:**
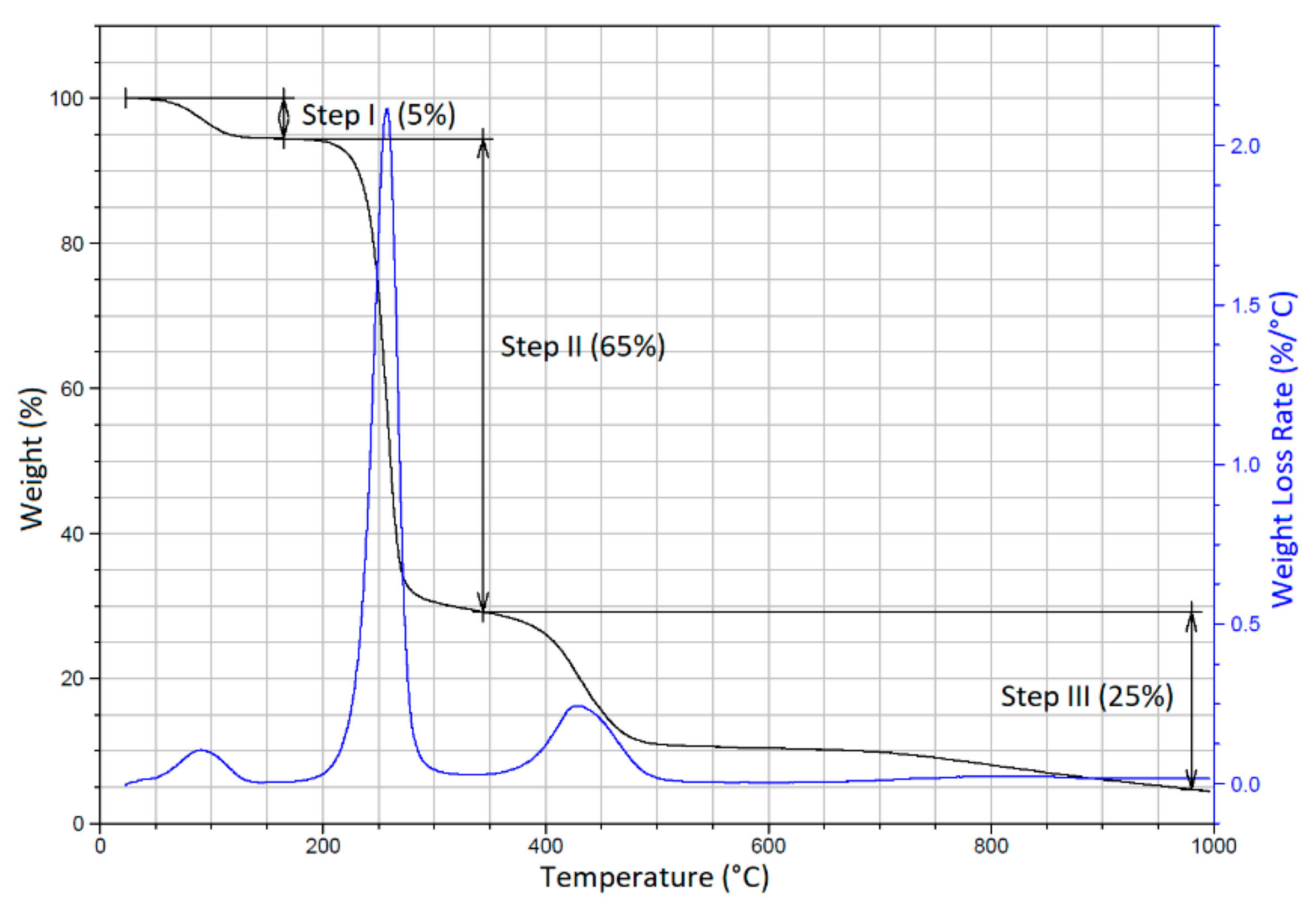
TGA and DTGA curves of the pure PVA.

Figure 7 shows the TGA and DTGA curves of the PVA sample with three pronounced degradation steps. Step I (25 °C–140 °C) obviously indicated the evaporation of water from the film (weight loss of ~5%). Step II (200–310 °C), with a weight loss of ~65%, was attributed to the dehydration of PVA accompanied by the generation of polyene structures. In Step III (350–530 °C), with a weight loss of ~25%, polyene structures were further transformed to low-molecular-weight products. [77,78,79]. Table 2 shows the initial and final temperatures of the degradation steps of PVA film and PVA-ND nanocomposite. 

**Figure 8:**
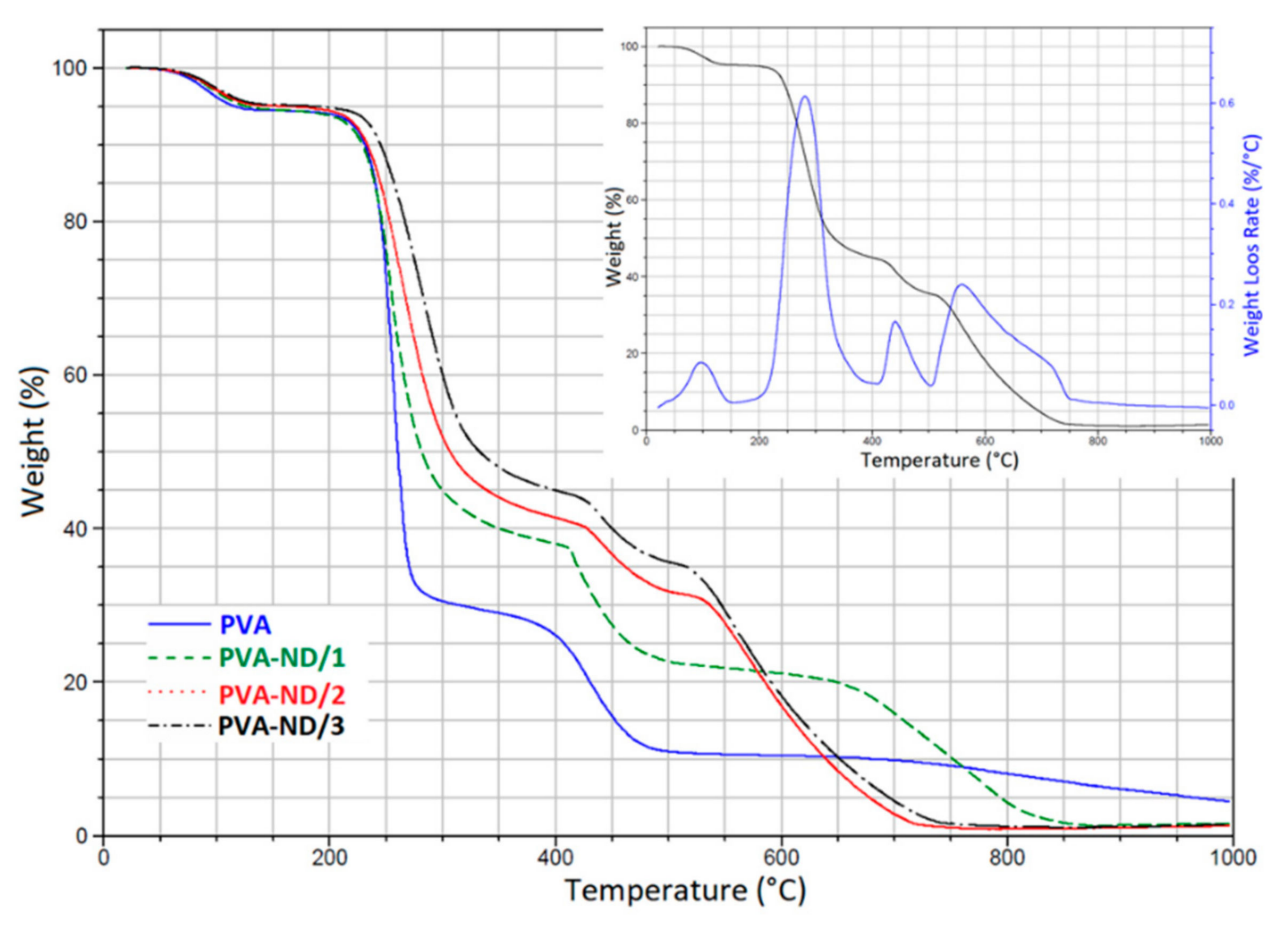
TGA thermograms of the pure PVA film and the PVA-ND nanocomposites; the inset graph shows the DTGA curve of the PVA-ND/3 nanocomposite (together with the original TGA curve).

The presence of NDs in the PVA structure clearly changed the degradation profile of the PVA-ND nanocomposites (Figure 8). Particularly, the three-step degradation diagram observed for PVA was changed to four steps for the PVA-ND nanocomposite films, as shown in the DTGA graph of the PVA-ND/3 nanocomposite (Figure 8 insert). The degradation was milder in the PVA-NDs nanocomposites than in the PVA, which was due to the incorporation of the ND nanoparticles into the PVA structure. The first stage of degradation occurred in the temperature range of ~30–145 °C owing to the evaporation of water. Stage two was from ~190 to 380 °C. The highest weight loss of 65% was seen for PVA-ND/1 (5% of the ND), and the lowest weight loss of 50% was observed for PVA-ND/3 (28% of the ND). The third step of decomposition of the PVA-ND nanocomposites could be observed at ~415–510 °C. The fourth decomposition stage was observed in the temperature range of ~515–750 °C for the PVA-ND/2 and PVA-ND/3 samples and of 630–870 °C for the PVA-ND1 sample. The fourth decomposition step was due to oxidative etching, where the carbon was oxidized to form carbon dioxide (CO_2_). The shift of the mentioned fourth decomposition stage to lower temperatures for the PVA-ND/2 and PVA-ND/3 samples was caused by a higher content of ND, which degraded totally around 570 °C in the pure state [61]. A comparison of TGA curves of the nanocomposites with that of pure PVA at high temperatures (above 500 °C) showed an interesting fact that ND acted as a catalyst of thermal decomposition of PVA in this temperature range. The nanocomposite samples were almost totally degraded at 870 °C in the case of PVA-ND/1 and at 750 °C in the case of both PVA-ND/2 and PVA-ND/3, while the pure PVA sample still showed a non-negligible residual weight even at 1000 °C. The residual weight at 1000 °C was ~1% for PVA-ND nanocomposites and ~5% for PVA. 

The weight loss of the PVA-ND films (Figure 8) was thus slowed down, compared to the pure PVA film (Steps II-III degradation). This might be explained first by the mere presence of ND that did not decompose in Step II and III, which thus reduced the heights of the two PVA decomposition steps, and second, the shift of Step II to higher temperatures could be attributed to the stabilization of the alcoholic groups of PVA against the dehydration reaction by the hydrogen bonding between the oxygen-containing functional groups of ND particles and the hydroxyl groups of PVA (Scheme 1). In the work of Santos et al. [21], it was suggested that the ND dispersion could act as a constraint for the diffusion of the evaporative degradation residues from the composites. Thus, TGA showed that the integration of ND particles changed the kinetics of thermal degradation of the PVA to some extent. However, note that all the films, including the pure PVA film, were usable up to ~230 °C, where their degradation begins.

### 3.5. DSC

**Figure 9:**
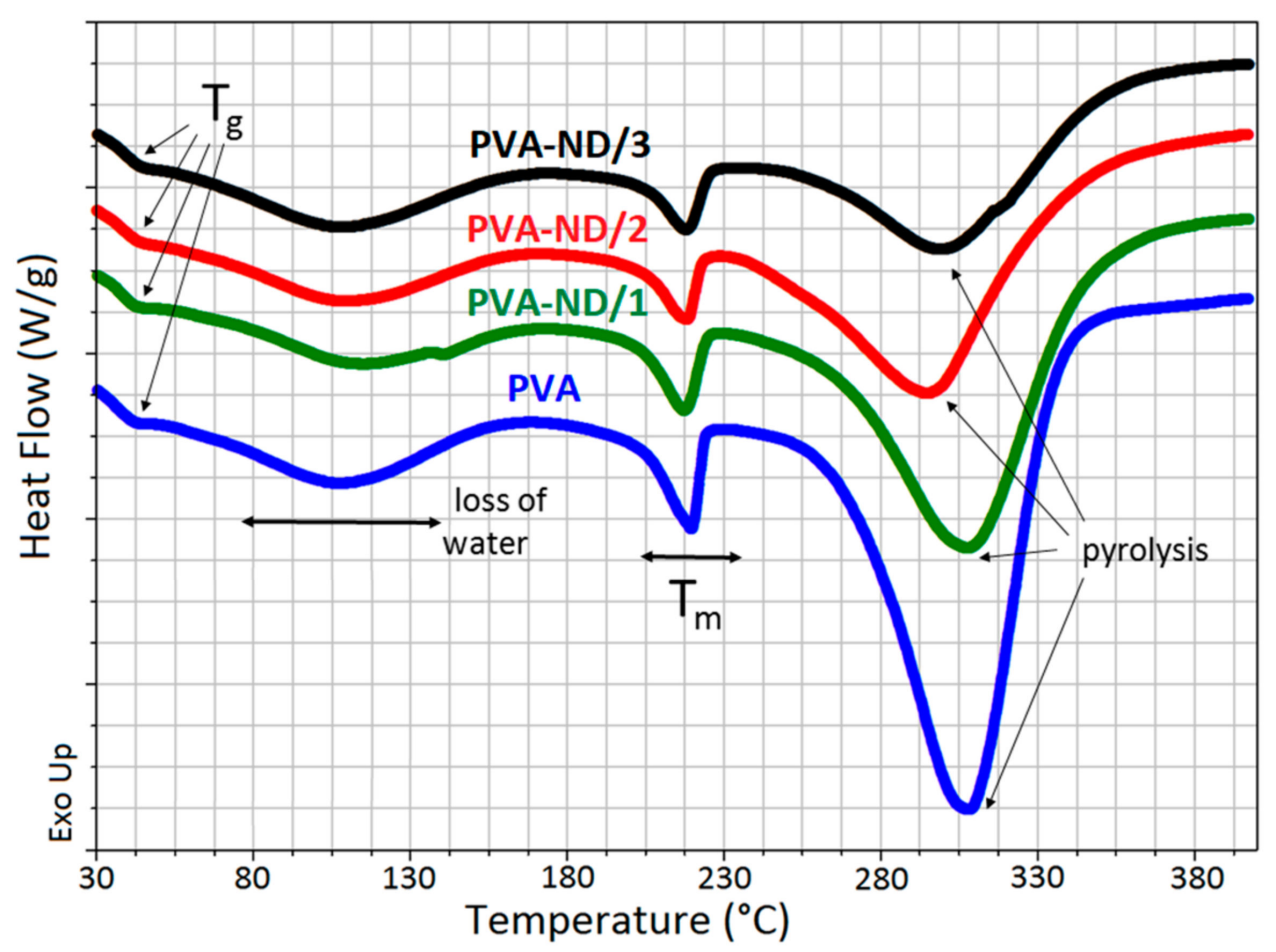
DSC curves of the pure PVA film and the PVA-ND nanocomposites.

Figure 9 shows the DSC heating curves for the PVA and PVA-ND samples with varying ND loadings. The PVA glass transition (*T_g_*) occurred in the temperature range from ~41 to 45 °C. The broad peak in the temperature range from ~60 to 170 °C was due to the release of water. The melting point of the PVA (*T_m_*) followed, indicated by a small, relatively sharp peak with a maximum at ~218 °C, which decreased in intensity with the increasing ND content in the samples. The rightmost large endothermic peak, in the temperature range from ~240 to 350 °C, represented the thermal pyrolysis of the PVA with a heat consumption of 690 J/g for PVA and 495, 347, and 260 J/g for PVA-ND/1, PVA-ND/2, and PVA-ND/3 nanocomposites, respectively. The *T_g_* increased slightly with the increasing amount of NDs in the PVA matrix, from 42 °C for PVA to 42.6 °C, 43 °C, and 43.8 °C for PVA-ND/1, PVA-ND/2, and PVA-ND/3, respectively. The increase in *T_g_* was probably caused by the partial crosslinking of the PVA by the NDs. The increase in *T_g_* correlated well with the DMA results, where an increase in *T_g_* was also observed.

The endothermic heat of fusion at ~218 °C was used to determine the crystallinity (*X_c_*) of PVA and the nanocomposites (Table 3). The *X_c_* of the polymers was determined from DSC curves using Equation (1), supposing a linear relationship between the endothermal peak area and the crystallinity:

(1) $$X_{c}=\frac{\Delta H}{\Delta H_{0}}\cdot \frac{1}{wt{\%}_{PVA}}\cdot 100\%$$

here, *X_c_* is the crystallinity of a semi-crystalline polymer (PVA), Δ*H* is the measured heat of fusion, Δ*H*_0_ is the heat required for melting of a 100% crystalline polymer; for PVA, it was 138.60 J/g [80], and *wt%_PVA_* is wt% of the PVA in the sample. The decrease in the *X_c_* (or Δ*H*) of PVA-ND nanocomposites corresponded with the increase in ND loading. After the incorporation of ND into the PVA matrix, the crystalline structure of PVA was disturbed to some extent, and the crystallinity somewhat decreased.

The results from the DSC measurements were in good agreement with the TGA measurements of the pristine PVA film and PVA-ND nanocomposites. The broad DSC peaks in the range from 60 °C to 150°C, due to the loss of water, correlated with Step I in the TGA (~45–160 °C). The large endothermic peak in the DSC scans in the range from ~240 to 350 °C correlated well with Step II in the TGA curves (~225–325 °C). The decrease in heat corresponding to the thermal pyrolysis of PVA/ND nanocomposite films (~240 to 350 °C) was caused first by the decrease in PVA weight percent in the samples alone because NDs did not degrade in this temperature range. However, the decrease was higher than only merely proportional to the PVA weight percent drop. This could be attributed to the reinforcement of the PVA structure with ND crosslinking (interactions of ND with the OH groups of PVA), which restricted the pyrolysis of PVA, in particular the PVA dehydration reaction.

### 3.6. Mechanical Properties

**Figure 10:**
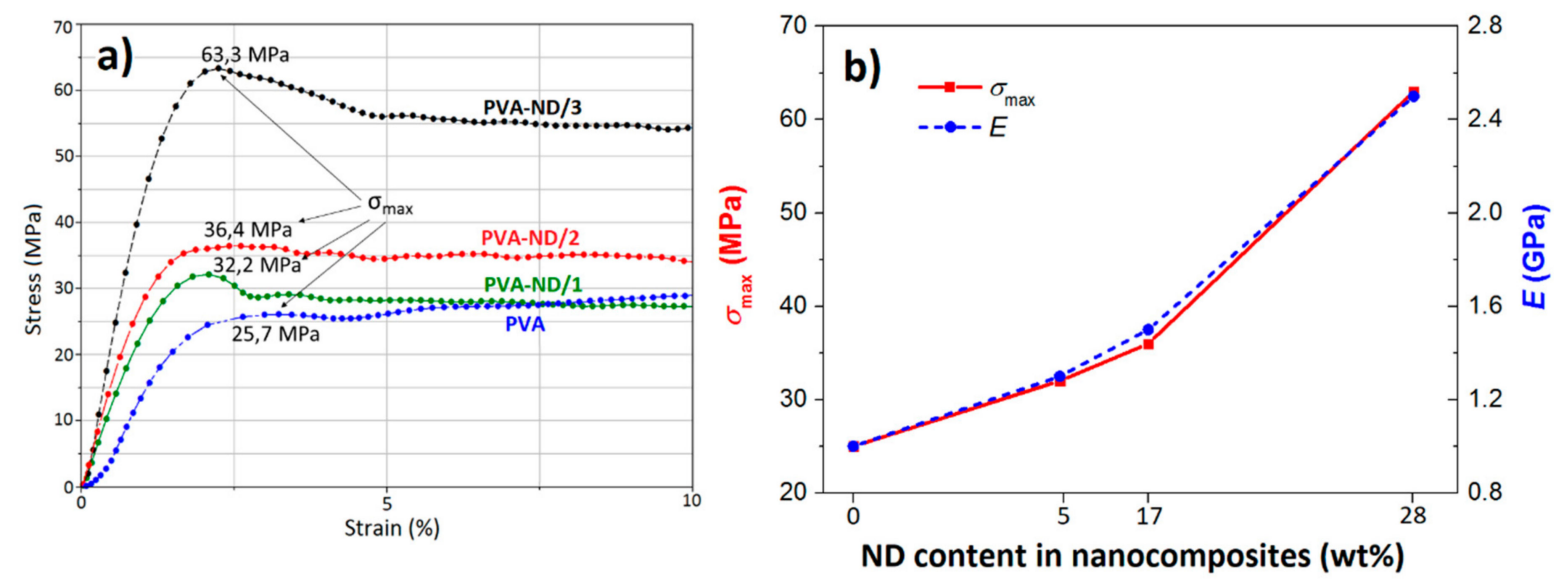
(**a**) σ-ε graph of the PVA and the PVA-ND nanocomposites and (**b**) dependence of the tensile strength (*σ_max_*) and Young’s modulus (*E*) on the ND content of the PVA-ND nanocomposites.

Figure 10a shows the tensile stress-strain curves of the PVA and PVA-ND films. It was observed that all samples behaved in a ductile manner: they showed a local maximum in the stress-strain curve, after which a yield behavior was observed. Li et al. [81] found that completely dried pure PVA films were brittle, i.e., exhibited brittle failure without yield and had a high tensile strength of around 100 MPa, while the presence of even a small amount of water made the films ductile (showing a yield behavior) and reduced substantially their tensile strength and modulus. These findings were not in contradiction with this work because, as the above TGA analysis showed, all the investigated films in this work contained a small amount of water of about 5 wt%. Taking into account this fact, our tensile stress-strain curve of the PVA film was relatively in good agreement with the literature [81]. 

The curves in Figure 10a clearly indicated that the tensile properties (strength, modulus, toughness) enhanced with an increasing amount of ND in the samples. Furthermore, the PVA-ND/3 film showed a substantially higher tensile strength (63.3 MPa) than both the PVA-ND/1 and PVA-ND/2 films (32.2 and 36.4 MPa, respectively). Figure 10b shows the relationship between the maximum tensile strength (*σ_max_*) and Young’s modulus (*E*), depending on the ND content in the PVA-NDs nanocomposites. The modulus of the PVA-ND/3 nanocomposite was found to be about 150% higher than in the case of pure PVA film. The high mechanical performance of the PVA-ND nanocomposites also showed that a good dispersion of the numerous small ND agglomerates, as mentioned in SEM, TEM, and SAXS/WAXS, together with a strong interaction between the PVA and NDs, was achieved. 

**Figure 11:**
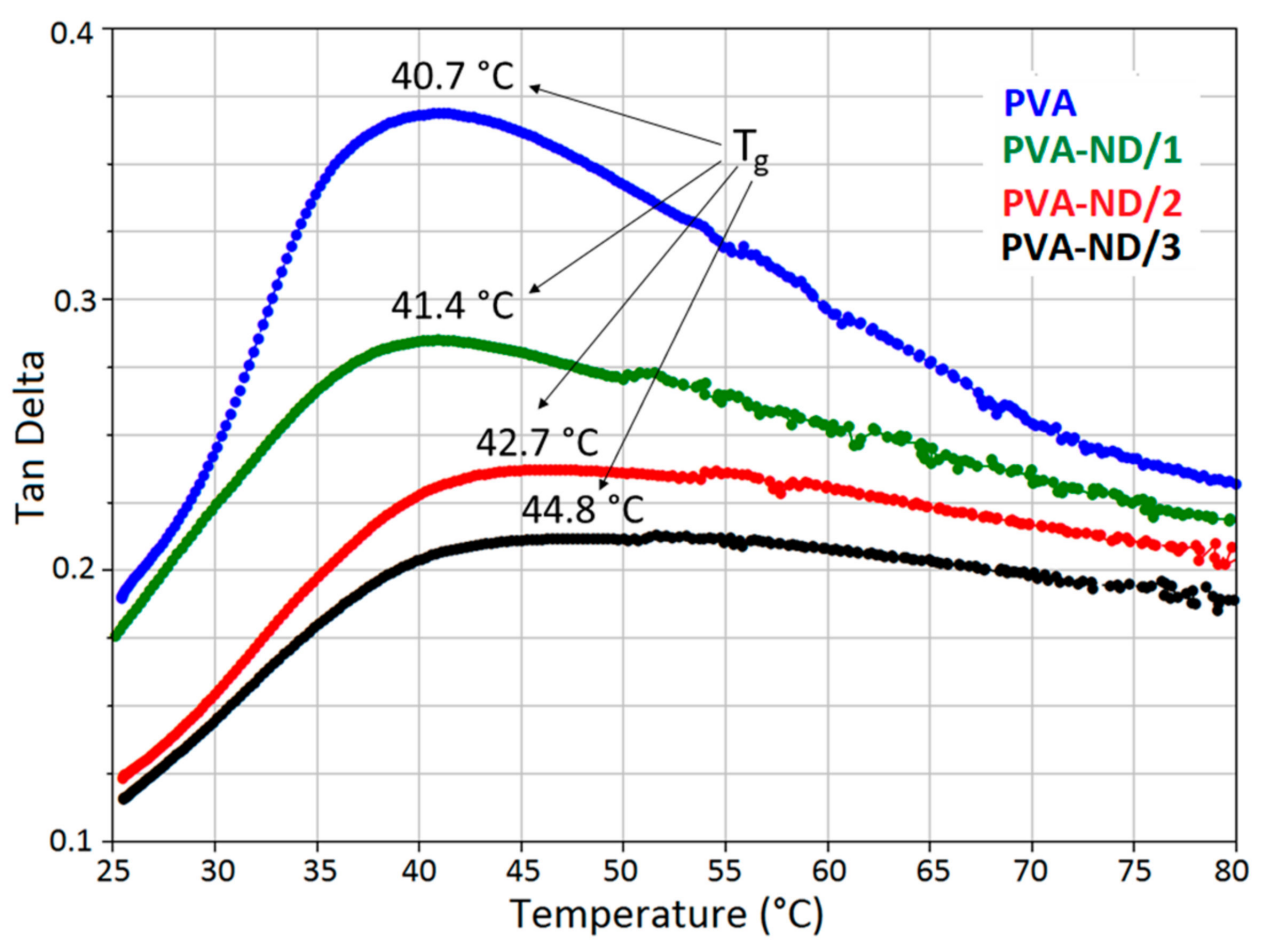
Mechanical properties and *T_g_* of the PVA and the PVA-ND nanocomposites.

From the dependence of tan delta on temperature (Figure 11), it was obvious that the *T_g_* slightly increased with increasing ND content. The observed *T_g_* values were 40.7 °C, 41.4 °C, 42.7 °C, and 44.8 °C for PVA, PVA-ND/1, PVA-ND/2, and PVA-ND/3, respectively. These values corresponded well with the *T_g_* values obtained from DSC (Table 3). The decrease in the intensity of the tan delta peaks with increasing ND content (Figure 11) indicated the increasing limitation of the mobility of the polymer chains due to the incorporation of the NDs. The results of the viscoelastic analysis of the nanocomposites corresponded well with the investigations of mechanical behavior in [82,83], proposing that the reinforcement PVA by the ND helped to restrict the molecular backbone movement.

## 4. Conclusions

In this research, PVA-based nanocomposites were prepared from dispersions with various ND loadings (5 wt% of PVA and 0.25, 1, and 1.9 wt% of ND) using a solution-casting method with the resulting ND contents in the composites being 5, 17, and 28 wt% in the completely dried state (however, all the investigated samples contained ~5 wt% of inbound water according to TGA). Raman spectroscopy, SAXS/WAXS, and FE-SEM showed that a successful dispersion of the ND agglomerates in the PVA matrix was achieved. FE-SEM showed that the agglomerates were several tens of nanometers in size, and SAXS indicated that the agglomerate structure did not significantly change with respect to their structure in the original dispersions. Raman spectroscopy, WAXS, and also DSC indicated that the formation of PVA nanocrystallites was restricted to some extent for samples with increasing ND content; however, no substantial change in the morphology of PVA was observed in WAXS. The main conclusion of this study was that the presence of ND led to an increased Young’s modulus and tensile strength of the PVA-ND nanocomposites indicating a good adhesion between the ND particles and the PVA matrix. The good adhesion was attributed mainly to the formation of hydrogen bonds between the oxygen-containing functional groups on the ND surface and OH groups of PVA. The highest tensile strength (~63 MPa) was detected for the PVA-ND/3 film with the highest ND content of 28 wt%. This represented an improvement by a factor of more than two with respect to the pure PVA film. It should be noted here that the films investigated in this work were not completely dried, but they contained about 5 wt% of inbound water, which has a substantial influence on the tensile properties of PVA-based materials (as described in Section 3.6). This should be kept in mind when comparing the values given in this work with literature. Both DSC and viscoelastic analysis showed that with an increasing amount of ND in the samples, a slight increase in glass transition temperature occurred. At the same time, the intensity of the tan delta peak decreased. Both these effects were attributed to the reinforcement of the polymer matrix with ND leading to restricted PVA chain mobility. Thus, due to the improved mechanical properties, these solution-cast PVA nanocomposites were a promising material for a variety of applications, including filter materials, batteries, fuel cells, and also for biomedical applications owing to the successful utilization of both PVA and NDs in medicine and pharmacology.

## Figures and Tables

**Table 1:** Composition of the vinyl alcohol-nanodiamonds (PVA-ND) dispersions and dried PVA-ND nanocomposite films.

Composite Designation	PVA—Dispersion (wt%)	NDs—Dispersion (wt%)	PVA—Film (wt%)	NDs—Film (wt%)
PVA	5	0	100	0
PVA-ND/1	5	0.25	95	5
PVA-ND/2	5	1	83	17
PVA-ND/3	5	1.9	72	28

**Table 2:** Initial and final temperatures of the degradation step of PVA-ND samples.

Composite Designation	T_I-ini_ (°C)	T_I-fin_ (°C)	T_II-ini_ (°C)	T_II-fin_ (°C)	T_III-ini_ (°C)	T_III-fin_ (°C)	T_IV-ini_ (°C)	T_IV-fin_ (°C)
PVA	25	140	200	310	350	530	-	-
PVA-ND/1	30	145	190	370	405	510	630	870
PVA-ND/2	30	140	190	380	415	510	515	750
PVA-ND/3	35	150	200	390	415	505	510	750

**Table 3:** *T_g_*, Δ*H,* and *X_c_* of the pure PVA film and the PVA in the PVA-ND nanocomposites.

	*T_g_* (°C)	*T_m_* (°C)	Δ*H* (J/g)	*X_c_* (%)
PVA	42.0	219.5	45.5	32.8
PVA-ND/1	42.6	217.1	38.5	29.2
PVA-ND/2	43.0	218.0	29.5	25.6
PVA-ND/3	43.8	217.8	29.3	29.4
